# Toward a statistical description of methane emissions from arctic wetlands

**DOI:** 10.1007/s13280-016-0893-3

**Published:** 2017-01-23

**Authors:** Norbert Pirk, Mikhail Mastepanov, Efrén López-Blanco, Louise H. Christensen, Hanne H. Christiansen, Birger Ulf Hansen, Magnus Lund, Frans-Jan W. Parmentier, Kirstine Skov, Torben R. Christensen

**Affiliations:** 1 Department of Physical Geography and Ecosystem Science, Lund University, Sölvegatan 12, 22362 Lund, Sweden; 2 Department of Bioscience, Arctic Research Centre, Aarhus University, Frederiksborgvej 399, 4000 Roskilde, Denmark; 3 Qatserisut 8, 3900 Nuuk, Greenland; 4 Arctic Geology Department, The University Centre in Svalbard, UNIS, P.O. Box 156, 9171 Longyearbyen, Norway; 5 Department of Geosciences and Natural Resource Management, University of Copenhagen, Øster Voldgade 10, 1350 Copenhagen K, Denmark; 6 Norwegian Institute of Bioeconomy Research, Høyskoleveien 7, 1430 Ås, Norway

**Keywords:** Emission, Greenland, Methane, Svalbard, Tundra

## Abstract

Methane (CH_4_) emissions from arctic tundra typically follow relations with soil temperature and water table depth, but these process-based descriptions can be difficult to apply to areas where no measurements exist. We formulated a description of the broader temporal flux pattern in the growing season based on two distinct CH_4_ source components from slow and fast-turnover carbon. We used automatic closed chamber flux measurements from NE Greenland (74°N), W Greenland (64°N), and Svalbard (78°N) to identify and discuss these components. The temporal separation was well-suited in NE Greenland, where the hypothesized slow-turnover carbon peaked at a time significantly related to the timing of snowmelt. The temporally wider component from fast-turnover carbon dominated the emissions in W Greenland and Svalbard. Altogether, we found no dependence of the total seasonal CH_4_ budget to the timing of snowmelt, and warmer sites and years tended to yield higher CH_4_ emissions.

## Introduction

The small coverage of measurement sites in arctic tundra causes large uncertainties in regional emission budgets of the greenhouse gas methane (CH_4_) (McGuire et al. 2012). The process-based upscaling of CH_4_ flux measurements requires detailed information about the local ground conditions (Davidson et al. 2016), which typically cannot be obtained with remote sensing techniques. Arctic tundra ecosystems are predicted to warm and change significantly in the near future (Johannessen et al. 2004; Callaghan et al. 2011a; Cohen et al. 2012), so there are pressing questions about the CH_4_ flux response to, e.g., earlier snowmelt and generally warmer growing seasons (Callaghan et al. 2011b). Temperature and water table position are often identified as key controls for the short-term CH_4_ fluxes (Turetsky et al. 2008, 2014; Tagesson et al. 2013), but longer-term seasonal patterns could relate more to the decomposability of the different pools of organic substrates and the development of plants (Christensen et al. 2003; Whalen 2005). So would an earlier snowmelt, causing a longer growing season, lead to larger seasonal emissions? If so, the potentially increased CH_4_ concentrations in the atmosphere could further amplify climate change effects.

Gas exchange measurements in the Arctic are challenging due to the harsh weather and logistical constraints. The used measurement techniques can also differ tremendously between sites (e.g., Crill et al. 1988; Wagner et al. 2003; Corradi et al. 2005; Parmentier et al. 2011), which complicates inter-site comparisons. The closed chamber technique has proven to be a robust method for CH_4_ flux measurements, but it is generally not applied continuously throughout the whole growing season (Olefeldt et al. 2013). In the larger framework of the Greenland Ecosystem Monitoring Program, three arctic sites were therefore equipped with the same automatic closed chamber system to continuously monitor CH_4_ fluxes on the same plots over many growing seasons and the subsequent freeze-in periods. The collected dataset gives unique possibilities to analyze the seasonal patterns at these different ecosystems. The first five years from one of the high-arctic sites (Zackenberg) were previously analyzed by Mastepanov et al. (2013). The derived flux pattern led the authors to hypothesize a bi-component origin of growing season CH_4_ emissions, driven by two different mechanisms related to slow and fast carbon turnover (Chanton et al. 1995). Accordingly, a first emission peak stems from the slow-turnover carbon of frost-damaged roots or cells of soil microorganisms (Skogland et al. 1988). As methane production from this carbon pool diminishes during the first months after snowmelt, the fast-turnover emissions from root exudates start to dominate the total emissions. This second component leads to a wide peak in the middle of the growing season that is related to the maturity of vascular plants and their root exudates (Ström et al. 2003). Similar interplays of the methanogenic carbon pools have been inferred from measurements in Canadian wetlands (Lai et al. 2014), and matching seasonal patterns have also been measured at Alaskan wetlands (Zona et al. 2016). Mastepanov et al. (2013) additionally reported a third component at the Zackenberg site during the autumnal freeze-in period. This final component is, however, most likely related to physical releases of stored gases in the soil rather than instantaneous methane production (Mastepanov et al. 2008; Pirk et al. 2015).

The formation and emission of CH_4_ can be studied using process-based models (Zhuang et al. 2004) or measurements of stable isotopic signatures (Hodgkins et al. 2015). The present study follows a different approach using a temporal separation of the flux time series to investigate the bi-component source pattern. We apply the method to measurements from three arctic sites located across a gradient from the low Arctic (central W Greenland) to the high Arctic (NE Greenland and Svalbard), and relate the resulting flux patterns to site differences of snow cover and ground thermal regime. Finally, we compare the total seasonal CH_4_ emissions with the length of the snow-free period and its overall temperature.

## Materials and methods

### Study sites

The site in Zackenberg Valley (74°30′N, 21°00′W) in the Northeast Greenland National Park lies in a high-arctic region with a mean annual air temperature of −9.9 °C (1958–1987), continuous permafrost, and a total annual precipitation of 286 mm on average (Hansen et al. 2008). Maximum snow depths vary interannually between 13 and 133 cm (Pedersen et al. 2016). The measurement site is located on the edge of a fen, on a large alluvial fan, whose vegetation is dominated by *Eriophorum scheuchzeri*, *Carex* cf. *stans*, *Dupontia psilosantha*, and moss species.

The Kobbefjord site (64°08′N, 52°23′W) in the Nuuk area in Western Greenland lies in a low-arctic fen, featuring a mean annual air temperature of −1.4 °C (1961–1990), no permafrost, and a total annual precipitation of about 750 mm (Cappelen 2013). Maximum snow depths in our measurement period varied between 120 and 150 cm interannually. The fen’s vegetation is dominated by *Scirpus caespitosus* and *E. angustifolium* (Bay et al. 2008).

**Fig. 1:**
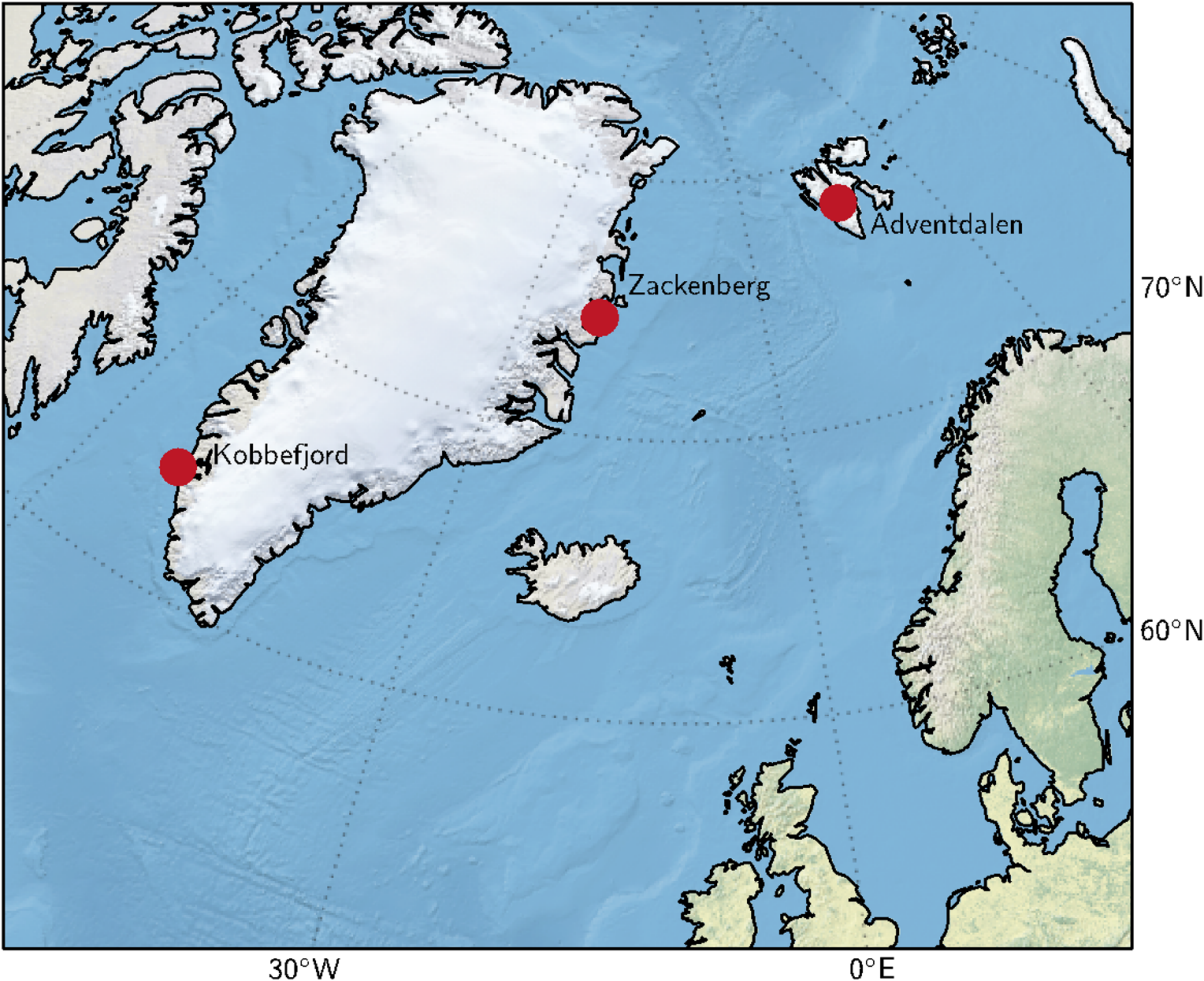
Site locations in the North Atlantic region

The site in Adventdalen Valley (78°11′N, 15°55′E) on Svalbard features low-centered ice-wedge polygons, which create fen conditions in the polygons (Christiansen 2005; Harris et al. 2009). The region’s mean annual air temperature is −6.7 °C (1961–1990), and the average total annual precipitation is 190 mm (Førland et al. 2012). The snow is largely redistributed by wind (Winther et al. 2003), leading to an average snow depth of about 20–30 cm at the site. The vegetation at this site features *Salix polaris* in drier spots, *E. scheuchzeri* and *Carex subspathacea* in wet locations, and moss species in usually inundated areas. Figure 1 shows the geographic location of the sites.

### Measurement setup

The three field sites are equipped with the same automatic chamber system based on Goulden and Crill (1997). A set of transparent chambers—each covering a square of 60 by 60 cm, with a height of 30 cm—are placed in close proximity to each other at each site. Kobbefjord and Adventdalen each have six chambers. The transect of six chambers at Zackenberg was extended by four additional chambers in 2011, so the most recent years feature ten instead of six chambers. Inside each chamber a fan ensures ventilation and gas mixing. High-density polyethylene tubes connect each chamber to the CH_4_ analyzer (Los Gatos Research, USA), which records CH_4_ concentrations at a rate of 1.0 Hz. The computer running these automatic measurements activates the chambers in succession for 10 min. During the first 3 min, the chamber is open for ventilation, and then closed for 5 min, and opened again for the last 2 min. Each chamber is activated once per cycle while the inactive chambers remain open. The flux calculation is based on ordinary least-squares regression as described by Pirk et al. (2016). This measurement setup yields CH_4_ flux time series with a resolution of 1 h from each chamber (2 h at Zackenberg after 2011). Our measurements typically start around the time of snowmelt and extend into the freeze-in period as long as the snow conditions allowed (typically covering June through October). Our dataset comprised 8 years of data from Zackenberg (2006–2015), 4 years from Kobbefjord (2012–2015), and 3 years from Adventdalen (2013–2015).

At Kobbefjord and Adventdalen, we used surface albedo measurements from a net radiometer in the direct vicinity of the chambers to determine the day of snowmelt. This day was defined as the first day with an average albedo of less than 0.3, which is a value typically matching our visual assessment. At Zackenberg, where there are no albedo measurements at the chambers, the day of snowmelt was determined by visual inspection on site when most snow had melted inside and around the chambers.

To further characterize each year, we calculated growing degree days (GDD) from daily minimum and maximum air temperatures (*T*) recorded by a nearby weather station. We used GDD = max (0, (*T*_min_ + *T*_max_)/2 − *T*_base_), with base temperature *T*_base_ = 0 °C.

### Temporal separation

The CH_4_ flux time series from each individual chamber was temporally separated into three components using the statistical mixing model technique. Two components of this temporal separation are intended to describe the different CH_4_ growing season sources hypothesized by Mastepanov et al. (2013), while the third component can capture potential autumn bursts (Mastepanov et al. 2008). This model used three Gaussian functions (each with three parameters, i.e., center position, width, and height) whose sum was optimized against the measured fluxes. We used the PyMix software package to estimate these parameters through the standard expectation–maximization algorithm which finds a maximum likelihood solution (Georgi et al. 2010). To reduce higher frequency variations and noise, daily medians of the measured fluxes were calculated first. The temporal separation was then applied from the first to the last day of measurements, between which potential measurement gaps were linearly interpolated. At Zackenberg and Kobbefjord, the data coverage of the daily flux time series was typically above 80%, while at Adventdalen about 50% of the time series needed to be gap-filled.

## Results

**Fig. 2:**
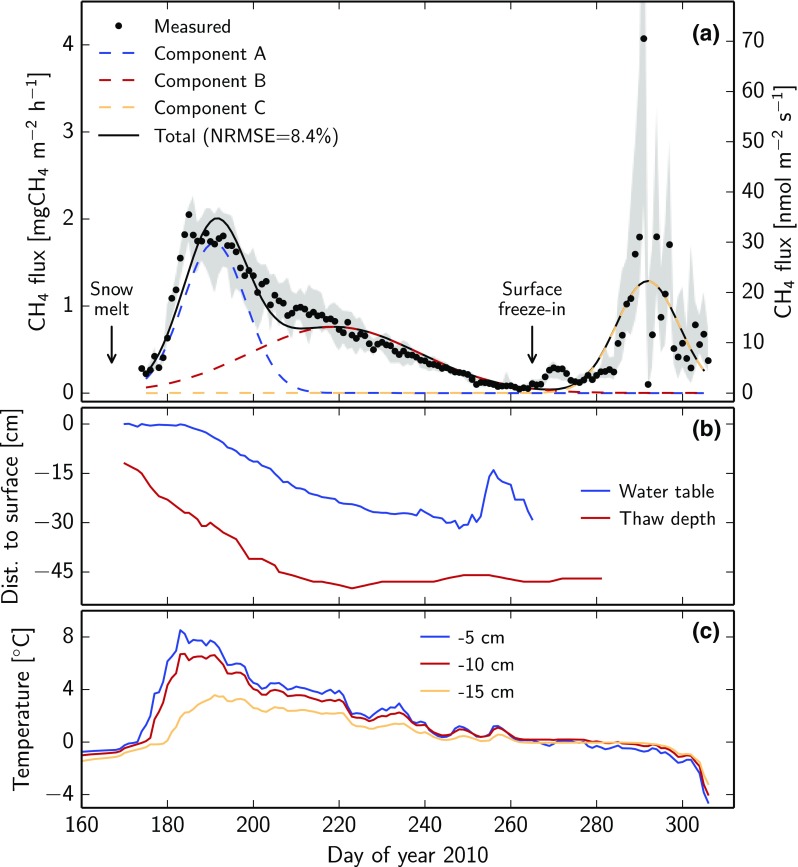
Example time series from Zackenberg, 2010. **a** CH_4_ flux from chamber 2. Measured points represent daily medians and the *shaded band* the 10–90 percentile range. **b** Water table and thaw depth with respect to the soil surface. **c** Soil temperature at three depths

Figure 2a shows an example of the measured CH_4_ fluxes together with the components of the temporal separation model for one chamber at Zackenberg, 2010. There was a steep rise of fluxes in the first month after snowmelt, which is largely attributed to the first Gaussian component A. Figure 2b shows that the thaw depth was increasing fastest during this period. The much wider component B describes the fluxes in the second half of the growing season. Finally, component C describes the autumn emissions during the freeze-in period, where large emission bursts occurred. As expected, the growing season fluxes show some agreement with abiotic factors like water table, thaw depth, and soil temperature, as shown in Fig. 2b, c. Individual measurement points can deviate from the model description, but the overall flux pattern appears to be well captured by the three components. Some deviations seem to relate to the symmetry of each of the used Gaussian peaks, indicating the limitations of the model descriptions (cf. “Discussion” section below). Despite these imperfections, the normalized root mean square error (NRMSE) of our model is typically found to be below 10%.

**Fig. 3:**
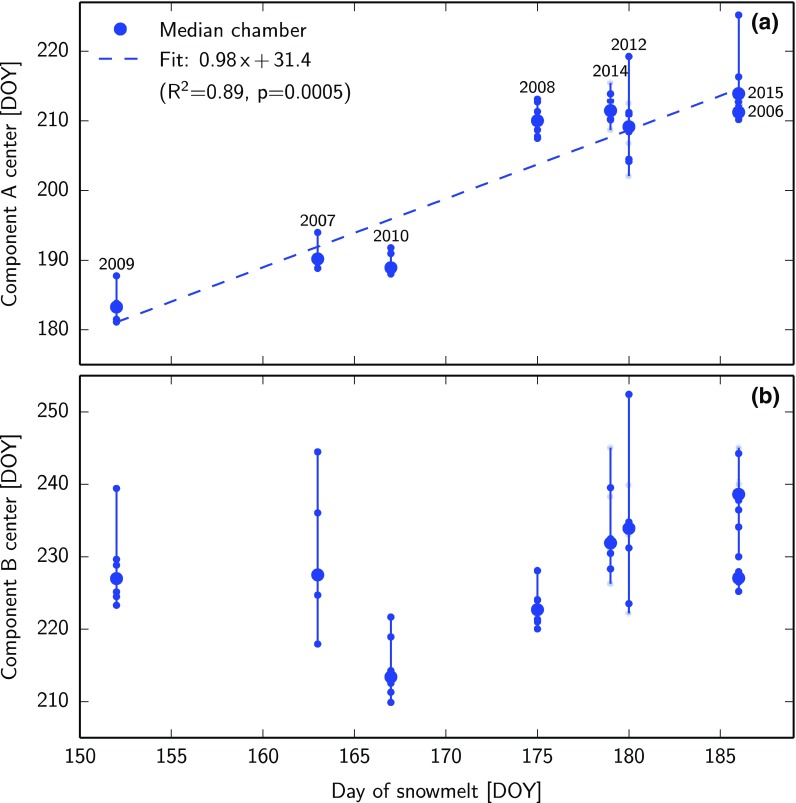
Timing of component centers versus day of snowmelt for individual chambers at Zackenberg. **a** Component A. **b** Component B. The *dashed line* shows the linear regression fit to the median of each year. The four additional chambers in years after 2011 are marked in a *lighter shade*

The timing of snowmelt at Zackenberg varied by more than one month over the years of our measurement campaign. We therefore investigated the relationship between the day of snowmelt and the flux pattern derived from our temporal separation. Figure 3a shows that there is a significant dependence of the center of component A (coinciding with the maximum growing season flux) to the day of snowmelt. The slope and intercept of the linear fit indicate that the maximum growing season flux typically occurred one month after snowmelt. Component B, on the other hand, had no clear dependence on the day of snowmelt, as shown in Fig. 3b. Its absolute position, however, corresponded well with the typically found maximum CO_2_ uptake fluxes at Zackenberg (around DOY 220, cf. “Discussion” section below), which is in line with the hypothesized origin of type B fluxes from root exudates related to plant development. The width of the Gaussian describing component A ranged between 5 and 14 days at Zackenberg. Component B was always found to be wider than A, ranging between 15 and 30 days.

**Fig. 4:**
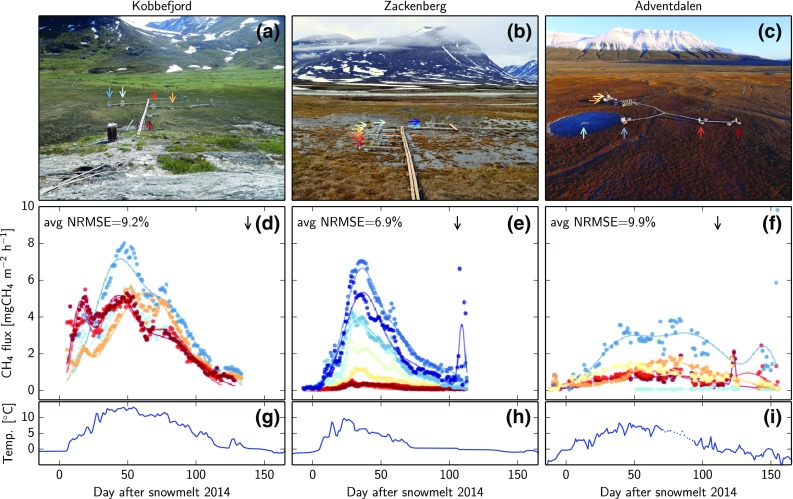
Site photos, fluxes, and soil temperatures. **a** Kobbefjord on July 14, 2015 (photo by Hanna Axén). **b** Zackenberg on July 4, 2012. **c** Adventdalen on October 8, 2015. **d**–**f** Corresponding flux measurements (*dots* representing daily medians) and temporal separation (*lines*) during the 2014 season with respect to day of snowmelt. Individual chambers are colored according to the *arrows* in the respective photo. *Black arrows* mark the beginning of the autumnal freeze-in period. **g**–**i** Soil temperatures at 10-cm depth. Temperatures shown as the *dotted line* at Adventdalen were taken from a different sensor, because data from the main sensor was not available

Figure 4 shows 2014’s flux data from all chambers at the three sites, with respect to the day of snowmelt. The differences between the chambers at each site were not random, but instead repeated the same inter-chamber ranking of flux magnitudes every year. To test the statistical significance of the inter-chamber differences, we performed a (repeated measures) analysis of variance (ANOVA) between all pairs of combinations at each site. While most inter-chamber differences were highly significant, every site also featured some combinations that did not have significantly different average CH_4_ fluxes. Across the sites, the maximum flux magnitude increased toward lower latitudes. Adventdalen, where daily fluxes were lowest on average, still featured a relatively long unfrozen period with a developed active layer. There was a small emission peak just before the day of snowmelt, as well as episodic bursts during the freeze-in period, similar to the autumn CH_4_ burst reported by Mastepanov et al. (2008).

The seasonal components suggested by the Zackenberg fluxes are not equally well detectable at Kobbefjord and Adventdalen. At Kobbefjord, the model found the components at sometimes quite different positions for each chamber (cf. Fig. 4d). At Adventdalen, the flux time series featured no distinct peaks to constrain the temporal separation model during the growing season (cf. Fig. 4f). Still, the mathematical description by the temporal separation captures the overall pattern of fluxes and the NRMSE is quite comparable to Zackenberg. Our temporal separation model is designed to use all three components to describe the measured fluxes. At Kobbefjord, however, component C cannot be mechanistically associated with the same processes as at the other two sites, because the flux measurements never continued into the autumnal freeze-in period. Besides, no permafrost is present, which is the hypothesized requirement for an autumn burst (Mastepanov et al. 2013).

**Fig. 5:**
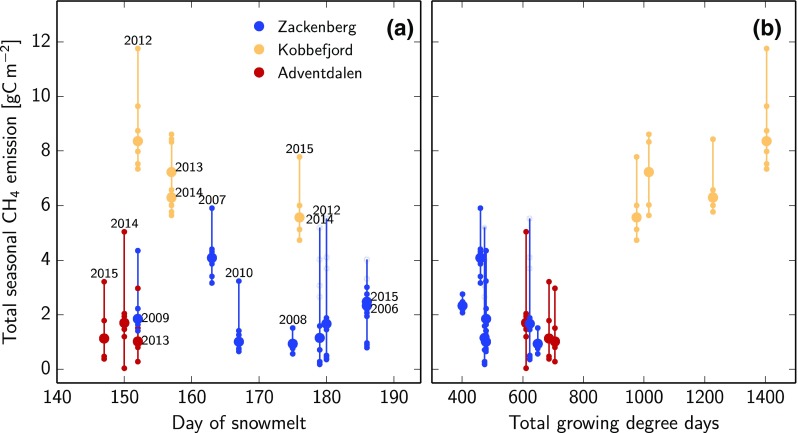
Total seasonal budget (1 June until 30 September) of each individual chamber with respect to day of snowmelt (**a**) and the total growing degree days of the respective year (**b**). The chamber representing the group’s median is marked with a *bigger circle*. The four additional chambers at Zackenberg in years after 2011 are marked in a *lighter shade*

Figure 5 shows the total CH_4_ budget between June 1 and September 30 of all chambers and years. Similar to the fluxes, there were large differences between the individual chambers at each site. Based on the median chamber budget, Zackenberg and Adventdalen typically yielded a similar seasonal budget of around 2 gC m^−2^, even though the flux patterns differed significantly (cf. Fig. 4e, f). Kobbefjord typically showed 2–3 times higher total emissions than Zackenberg and Adventdalen, and is the only site where total growing degree days appear to affect the total seasonal budget. Due to the limited number of measured years, however, this dependence is not significant. No clear relationship between the total seasonal CH_4_ budget and day of snowmelt was found within or across sites. However, the individual sites formed clusters with respect to total growing degree days, indicating a trend of higher CH_4_ emissions at warmer sites and years.

## Discussion

We used the hypothesized bi-component origin of CH_4_ emissions in relation to the timing of snowmelt to describe flux patterns at different arctic tundra sites. Finding the statistical distributions of the seasonal flux patterns using only the day of snowmelt is useful to explore site differences and potentially upscale fluxes to areas where no measurements exist. The underlying hypothesis based on slow and fast-turnover carbon could not be tested with our dataset, so alternative explanations remain possible. For example, CH_4_ fluxes could originate from one dominating source, and surface fluxes could primarily be a result of the seasonal patterns of soil temperature and water table rather than differences in substrate. The sharp rise of emissions after snowmelt at Zackenberg could also stem from stored organic acids or gases that were produced in the previous summer. Many such mechanisms could, however, still be captured with the temporal separation model proposed here or a variation thereof. Therefore, even when the underlying drivers are different, our statistical breakdown would remain useful to describe spatial and temporal patterns in CH_4_ fluxes. While Mastepanov et al. (2013) treated individual chambers as replicates and derived the flux pattern from mean values and standard deviations, the present study analyzed each chamber individually using a temporal separation into three-components. The choice of Gaussian functions for each component was intended to give a simple description with a minimal number of fitting parameters, so the model did not explicitly represent the mechanisms underlying the suggested hypothesis. Therefore, this model may not resolve the exact shape and integral of the individual seasonal components, as indicated by the mismatches seen in Fig. 2. However, the model’s simplicity leads to numerically robust and intuitive results with three clearly distinguished peaks, which allowed us to investigate the peak center positions at Zackenberg. The results indicate a significant dependence between the timing of the first peak (component A) and day of snowmelt with an average lag of 31.4 days, supporting the findings by Mastepanov et al. (2013). The timing of the second peak (component B)—hypothesized to stem from root exudates of plants—was independent of the day of snowmelt. The center of component B occurred between approximately DOY 210 and 240 (cf. Fig. 3b), which coincides with the typical time of maximum CO_2_ uptake fluxes in this wetland (Nordstroem et al. 2001; Mastepanov et al. 2013). This match further supports the bi-component hypothesis, because root exudates are expected to correlate with plant growth as measured by CO_2_ fluxes (Ström et al. 2003). In a nearby heath ecosystem, the plant dynamics later in the growing season have been shown to depend more on incoming sunlight than the timing of snowmelt (Lund et al. 2012). Therefore, a strong dependence between component B and the day of snowmelt is not expected, which follows our hypothesis. After confirming the earlier findings at the Zackenberg site, the same analysis was performed on data from the two sites at Kobbefjord and Adventdalen. The fluxes at these sites, however, showed no clear presence of three seasonal components, so a correlation of the first component with the date of snowmelt did not exist. These differences between the three sites suggest quite different dominating processes behind the CH_4_ emissions. At Kobbefjord, for example, the growing season CH_4_ flux has one strongly expressed component (cf. Fig. 4d), which bears a resemblance to component B because of its large width. This site features no permafrost, so despite the seasonal ground freezing, the physical mechanisms proposed to lie behind the autumn CH_4_ burst cannot be at work (Mastepanov et al. 2008, 2013; Pirk et al. 2015). However, we cannot fully exclude the presence of this flux component at Kobbefjord, because our measurements never continued long enough into the freeze-in period (November–December). Component A might well be present at this site, but could be masked by a shoulder of the much larger component B. The dominance of the component B would be in line with the finding that the total growing season CH_4_ emission at Kobbefjord appears linearly related to the total growing degree days per season (cf. Fig. 5b). At Zackenberg, in contrast, this relation cannot be seen, possibly because component A is much more pronounced than it is at Kobbefjord.

At Adventdalen, where permafrost is present, the CH_4_ autumn burst (flux component C) was observed, as suggested by the physical mechanism. Similar autumnal flux patterns with significant contributions to the CH_4_ budget were reported from permafrost-underlain tundra in Alaska (Sturtevant et al. 2012; Zona et al. 2016). The short observation history in Adventdalen and gaps in the data, however, prevent a detailed analysis of this peak, leaving this task for future studies. Flux component A was either small or irregular compared to Zackenberg, which could be related to climatic differences during wintertime. At Zackenberg, despite the proximity to the sea (which is ice-covered for a large part of the year), the climate is stable and continental. Wintertime air temperature typically varies between −10 and −30 °C. In combination with the relatively thick snow cover of up to 1.3 m at the site (Pedersen et al. 2016), the harsh conditions lead to a constant soil temperature, which is low enough to suppress microbial decomposition processes until the soil starts to thaw (around the day of snowmelt). Adventdalen, on the other hand, has a maritime climate with changeable weather in wintertime. Air temperature can rise above 0 °C in episodic warm spells in the autumn, winter, and spring. Together with the relatively thin snow cover of about 20–30 cm, which can melt and refreeze repeatedly, this leads to a strongly varying soil temperature and episodic warming of the top of the permafrost. CH_4_ attributed to type A decomposition, therefore, may have escaped to the atmosphere before complete snowmelt in May, and was not captured by our measurements. Thus, flux component B is predominant at this site during the growing season. Furthermore, due to the polygonal ground pattern in Adventdalen and the associated differences in soil wetness, the flux magnitude varies strongly on small spatial scales. The overall interannual temperature variations as quantified by the total growing degree days are relatively small (cf. Fig. 5b) and explain little of the interannual variations of CH_4_ emissions.

Arctic winter precipitation is both observed and projected to change with climate warming, affecting snow cover differently depending on the season and region within the Arctic (Callaghan et al. 2011b; Derksen and Brown 2012). Arctic coastal regions (such as our sites) are likely to experience strong decreases of snow cover duration due to an earlier snowmelt in spring (Callaghan et al. 2011b), which would prolong the growing season. At each of our three sites, there was no indication that an earlier snowmelt would increase the total seasonal amount of emitted CH_4_ (cf. Fig. 5a). This finding is in line with Oberbauer et al. (1998), who observed no statistically significant difference in total CH_4_ emissions in a snow removal experiment in Alaskan tundra. Variations of the wintertime snow thickness, on the other hand, were found to increase CH_4_ emission, largely as a response to soil warming (Blanc-Betes et al. 2016). So it could be argued that the shorter growing season could be compensated by typically higher soil temperature (higher CH_4_ fluxes) in years with a thick, long-lasting snowpack (Stiegler et al. 2016). Reciprocally, an earlier snowmelt can in part be due to less wintertime precipitation, which in turn leads to a lower water table position upon melt in summertime and therefore lower CH_4_ fluxes. Note, however, that these interannual differences can in the long term be overruled by climate warming and potential permafrost thawing, which is expected to increase both CH_4_ and CO_2_ emissions (Schädel et al. 2016).

## Conclusions

The CH_4_ emission patterns differed strongly between the three measurement sites. The Zackenberg site featured two clearly distinct flux components during the growing season, which responded differently to the timing of snowmelt, as expected from the bi-component hypothesis. Zackenberg and Adventdalen showed a third component during the autumnal freeze-in period, which is presumably caused by physical mechanisms in permafrost regions (Pirk et al. 2015). The absence of such large CH_4_ autumn bursts at Kobbefjord, where seasonal ground freezing without permafrost occurs, remains to be investigated in future studies with measurements covering this period. To further investigate the origin of the different flux components, future studies could aim to measure the isotopic signature of the CH_4_ source to resolve potential differences between components A, B, and C. From the present study, we expect more distinct source variations at Zackenberg than at Kobbefjord and Adventdalen, where growing season emissions appear dominated by one single component (B). Another approach could involve laboratory studies with soil samples from the different sites, which could be subjected to freeze–thaw cycles to study type A fluxes. With a better understanding of the different components, the modeling of CH_4_ fluxes using the specifics of the underlying processes can be improved. Our statistical analysis of CH_4_ emission from arctic wetlands can be used to predict the temporal flux patterns based on a minimal amount of information, namely the timing of snowmelt. Across the sites and years, the seasonality of the flux patterns was related to the timing of snowmelt, which did, however, not significantly affect the total seasonal budget.
